# Mechanistic and Single-Dose *In Vivo* Therapeutic Studies of Cry5B Anthelmintic Action against Hookworms

**DOI:** 10.1371/journal.pntd.0001900

**Published:** 2012-11-08

**Authors:** Yan Hu, Bin Zhan, Brian Keegan, Ying Y. Yiu, Melanie M. Miller, Kathryn Jones, Raffi V. Aroian

**Affiliations:** 1 Section of Cell and Developmental Biology, University of California San Diego, La Jolla, California, United States of America; 2 Section of Tropical Medicine, Department of Pediatrics, Baylor College of Medicine, Houston, Texas, United States of America; McGill University, Canada

## Abstract

**Background:**

Hookworm infections are one of the most important parasitic infections of humans worldwide, considered by some second only to malaria in associated disease burden. Single-dose mass drug administration for soil-transmitted helminths, including hookworms, relies primarily on albendazole, which has variable efficacy. New and better hookworm therapies are urgently needed. *Bacillus thuringiensis* crystal protein Cry5B has potential as a novel anthelmintic and has been extensively studied in the roundworm *Caenorhabditis elegans*. Here, we ask whether single-dose Cry5B can provide therapy against a hookworm infection and whether *C. elegans* mechanism-of-action studies are relevant to hookworms.

**Methodology/Principal Findings:**

To test whether the *C. elegans* invertebrate-specific glycolipid receptor for Cry5B is relevant in hookworms, we fed *Ancylostoma ceylanicum* hookworm adults Cry5B with and without galactose, an inhibitor of Cry5B-*C. elegans* glycolipid interactions. As with *C. elegans*, galactose inhibits Cry5B toxicity in *A. ceylanicum*. Furthermore, p38 mitogen-activated protein kinase (MAPK), which controls one of the most important Cry5B signal transduction responses in *C. elegans*, is functionally operational in hookworms. *A. ceylanicum* hookworms treated with Cry5B up-regulate p38 MAPK and knock down of p38 MAPK activity in hookworms results in hypersensitivity of *A. ceylanicum* adults to Cry5B attack. Single-dose Cry5B is able to reduce by >90% *A. ceylanicum* hookworm burdens from infected hamsters, in the process eliminating hookworm egg shedding in feces and protecting infected hamsters from blood loss. Anthelmintic activity is increased about 3-fold, eliminating >97% of the parasites with a single 3 mg dose (∼30 mg/kg), by incorporating a simple formulation to help prevent digestion in the acidic stomach of the host mammal.

**Conclusions/Significance:**

These studies advance the development of Cry5B protein as a potent, safe single-dose anthelmintic for hookworm therapy and make available the information of how Cry5B functions in *C. elegans* in order to study and improve Cry5B function against hookworms.

## Author Summary

Hookworm infections are one of the great parasitic diseases of our time, infecting more than half a billion people worldwide and are a significant source of iron-deficient anemia. Although mass drug administrations to eliminate hookworms from children and pregnant women are being deployed, all the drugs for treatment we have lack full potency against the parasites and are showing signs of reduced efficacy. Crystal proteins, like Cry5B, made by *Bacillus thuringiensis* are as a class considered safe to vertebrates and have been shown to have efficacy against intestinal roundworms like hookworms. Here we show that the key mechanistic details of how Cry5B functions in hookworms is conserved with that of the model free-living roundworm *Caenorhabditis elegans*, which has implications for confirming Cry5B safety in vertebrates and for enhancing Cry5B efficacy against roundworms. Furthermore, we show that Cry5B works effectively as a single-dose drug against hookworm infections in hamsters and can be formulated to increase its efficacy, eliminating 97% of the parasites in a single dose. These results advance the development of a novel, safe single-dose therapy for hookworm infections in humans.

## Introduction

Hookworms (*Ancylostoma duodenale*, *Necator americanus*, and, less commonly, *Ancylostoma ceylanicum*) are major soil-transmitted helminths (nematodes, roundworms) that parasitize humans, infecting 576–740 million people globally and are the leading source of iron-deficient anemia in endemic areas [1]. Hookworms are estimated by some to be second only to malaria in terms of disease burden by a parasite ([2], [3] where they are associated with ∼22 million disability adjusted life years). Hookworm infections lead to blood loss and anemia that most significantly impacts children and pregnant women leading to growth and cognitive stunting in children and increased risk of death or low birthweight babies during pregnancy. For mass drug administration against soil-transmitted helminths like hookworms, the current drug of choice is albendazole [4]. The single-dose cure rate with albendazole for hookworm infection is variable with an average of about 70%, leaving clear room for improvement [5]. More worrisome are increasing numbers of reports with lower efficacies of albendazole and possible resistance [6], [7]. Resistance to the class of drugs to which albendazole belongs is already rampant in veterinary medicine [8], [9]. Past experiences with infectious diseases (*e.g.*, malaria) has taught us that reliance on one drug for treatment of hundreds of millions of infected peoples inevitably leads to treatment failure.

One promising group of alternative anthelmintics is roundworm-active crystal proteins, in particular Cry5B, made by *Bacillus thuringiensis* [10]. Three 14 mg/kg Cry5B doses administered once/day for three days resulted in an 89% reduction in *Ancylostoma ceylanicum* hookworm burdens in hamsters [11]. *A. ceylanicum* is a zoonotic hookworm species, closely related to the major human parasite *Ancylostoma duodenale*, and is emerging as an important human parasite in Southeast Asia [12]. *A. ceylanicum* infections in hamsters are also considered a good model for hookworm disease in humans [13]. Cry5B is also effective against *Heligmosomoides bakeri* (*polygyrus*) roundworm infections in mice as a single 90 mg/kg dose was able to reduce parasite burdens by 70% [14]. It was furthermore shown that Cry5B rapidly degrades in simulated gastric fluids, suggesting that protection of Cry5B from gastric fluids should increase its efficacy [14].

The response of the free-living roundworm *Caenorhabditis elegans* to Cry5B has been extensively studied and analyzed, including via genetic screens, microarray analyses, whole-genome RNAi analyses, and characterization of innate immune responses [15], [16], [17], [18], [19], [20], [21]. Cry5B has been demonstrated to function as a pore-forming protein that intoxicates via binding to invertebrate-specific glycolipid receptors on the *C. elegans* intestine [16], [22], [23], [24]. One of the key innate immune pathways activated in *C. elegans* by Cry5B is the p38 mitogen activated protein kinase (MAPK pathway), evidenced by increased phosphorylation of *C. elegans* p38 upon treatment with Cry5B [16], [20]. Activation of the p38 MAPK pathway is important for *C. elegans* since loss of the pathway via genetic mutation or knock-down RNA interference (RNAi) results in >100-fold hypersensitivity of *C. elegans* to Cry5B [16], [21]. Although the mechanism of Cry5B action in *C. elegans* has been extensively studied, there has been no work in parasitic roundworms apart from the finding that Cry5B is able to bind glycolipids isolated from the hookworm *Ancylostoma ceylanicum* [11].

Here we investigate whether the mechanism of action of Cry5B in *A. ceylanicum* hookworms is conserved with its mechanism of action in *C. elegans*. We furthermore investigate whether single-dose Cry5B (*vs.* three doses over three days previously reported) can have efficacy against hookworm infections since single-dose therapy is the standard for mass drug administration against hookworms. Finally we test whether a simple formulation designed to neutralize acidic gastric fluid might increase Cry5B efficacy against hookworm infections *in vivo*.

## Materials and Methods

### Medium reagents or solutions

Reagents for hookworm culture medium (HCM): RPMI 1640, Fetal Bovine Serum (FBS), Penicillin-Streptomycin and Fungizone Antimycotic were all purchased from Gibco, U.S.A. Reagents for western blot: anti-α-tubulin antibody produced in mouse was purchased from Sigma-Aldrich, USA; Phospho-p38 MAPK (Thr180/Tyr182) Rabbit mAb was bought from Cell Signaling Technology, USA); Goat anti-rabbit IgG-HRPand Goat anti-mouse IgG-HRP were purchased from Santa Cruz Biotechnology, Inc. The other reagents: SB203580 (p38/RK MAP Kinase Inhibitor) was purchased from InvivoGen, USA. Glucose and Galactose were purchased from Sigma-Aldrich, USA. Hanks' Balanced Salt Solution (HBSS) (pH 7.2) was purchased from Gibco, USA.

### Animals

Three to four-week-old male Golden Syrian hamsters (HsdHan:AURA) were purchased from Harlan Laboratories and were infected at approximately 4–5 weeks of age with ∼150 infectious *A. ceylanicum* larvae [11], [25]. Hamsters were provided with food and water (*ad libitum*). All animal experiment was carried out under protocols approved by either the UCSD or the Baylor College of Medicine Institutional Animal Care and Use Committees (IACUC). All housing and care of laboratory animals used in this study conform to the NIH Guide for the Care and Use of Laboratory Animals in Research (see 18-F22) and all requirements and all regulations issued by the United States Department of Agriculture (USDA), including regulations implementing the Animal Welfare Act (P.L. 89-544) as amended (see 18-F23).

### Preparation of purified Cry5B

Cry5B was produced and purified as described [24] and suspended either in water/150 mM Tris buffer (pH 8.5) for *in vivo* experiments or dissolved in 20 mM HEPES (pH 8.0) for *in vitro* experiments.

### Mechanism of Cry5B action against *A. ceylanicum*

Hamsters were orally infected with *A. ceylanicum* larvae and euthanized at 17–20 days post-infection (P.I.). The intestines were removed from the animals, opened, and incubated in 37 C pre-warmed Hank's buffer in a 37°C, 5% CO_2_ incubator for an hour to free the worms from intestine [26]. Healthy and energetic worms were picked, washed with RPMI with antibiotics (100 U penicillin/100 µg/mL streptomycin and 10 µg/mL Fungizone), and cultured in HCM [11]. For the *in vitro* studies of galactose protection of hookworm adults from Cry5B intoxication, 6 groups of 10 hookworms were placed in wells of a 24-well microtiter plate (5 females and 5 males per well) containing either HCM alone or HCM supplemented 100 mM glucose, 100 mM galactose, 10 µg/mL Cry5B, 100 mM glucose+10 µg/mL Cry5B, or 100 mM galactose+10 µg/mL Cry5B (500 µL final volume in each well). Pictures were taken after 48-h or 96-h incubation in a 37°C CO_2_ incubator. For the study of the role of p38 MAPK kinase pathway in *A. ceylanicum* response to Cry5B, four groups of 10 hookworm adults (5 female and 5 males per well) were placed in wells of a 24-well plate containing HCM and incubated with or without 50 µM SB203580 for 24 hr in a 37°C CO2 incubator. The worms were then treated with/without 1 µg/mL Cry5B and incubated for another four days. Pictures were taken 96 hr later with an INFINITY1 camera. Results shown are representative of three independent trials for each of these experiments with 8–10 worms/condition/trial. For all *in vitro* experiments, worm health was assessed based upon motility, morphology, and shape relative to healthy controls.

### p38 MAPK immunoblotting

Five hookworm adults (mix gender) were incubated at 37°C in a CO2 incubator in HCM with or without 100 µg/mL Cry5B for 1 hour, and then the worms were picked to 1.5 mL microfuge tube with 50 µL of 1× sodium dodecyl sulfate loading buffer. The worms were boiled for 10 mins and 25 µL of lysate were used for immunoblotting. Monoclonal antibody to phospho p38 MAPK was used at 1∶500 and monoclonal antibody to α-tubulin was used at 1∶3000 [20]. Results shown are representative of three independent trials. Western blots were otherwise carried out as indicated [20].

### Single dose of Cry5B curative experiment

Forty-two 4-week-old male Golden Syrian hamsters (*Mesocricetus auratus*) were each orally infected with ∼150 third-stage larvae (L3) of *A. ceylanicum*. Sixteen days P.I., the infected hamsters were divided into 6 groups (7 hamsters each), and each group of hamster was orally administered with single dose of either 1 mg, 3 mg, 10 mg of Cry5B resuspended in distilled water, 3 mg of Cry5B in 150 mM Tris, pH 8.5, water (control), or 150 mM Tris, pH 8.0 (control), in a total volume of 0.5 ml. Feces from each group of hamsters were collected (overnight day 20–21 P.I.) and the fecal egg counts were performed by using a McMaster chamber (Hausser Scientific, Horsham, PA). After collecting feces day 21 P.I., the hamsters were euthanized and adult hookworms were collected from the small intestine of each hamster. Body weight was monitored (body weights just prior to treatment are shown in Figure S1) and about 100 µl of blood was collected from the lateral saphenous vein of each hamster before and after treatment with Cry5B. The collected blood was used to measure hemoglobin by using Hemocue HB201^+^ (Hemocue, Angelholm Sweden). Numerical values from this experiment (*i.e.*, intestinal worm burdens, eggs per gram of feces, hemoglobin levels) are given in Table S1.

### Statistical analysis

Data analysis of intestinal worm burdens and fecal egg counts was plotted using Prism 5 (GraphPad Software Inc., La Jolla, CA, U.S.A.). For worm burdens, average indicates the average worm burdens amongst all the hamsters in each treatment group. For fecal egg counts, average indicates the egg count per group from all cages in the group at a given time point. Statistical comparisons were carried out using one-way ANOVA and Tukey's Honestly Significant Difference test (JMP version 10), except for a direct comparison between the 3 mg treatment groups (water *vs.* Tris buffer), which was via one-tailed student t-test assuming unequal variances.

## Results

### Conservation of mechanism of Cry5B action between hookworms and *C. elegans*

**Figure 1:**
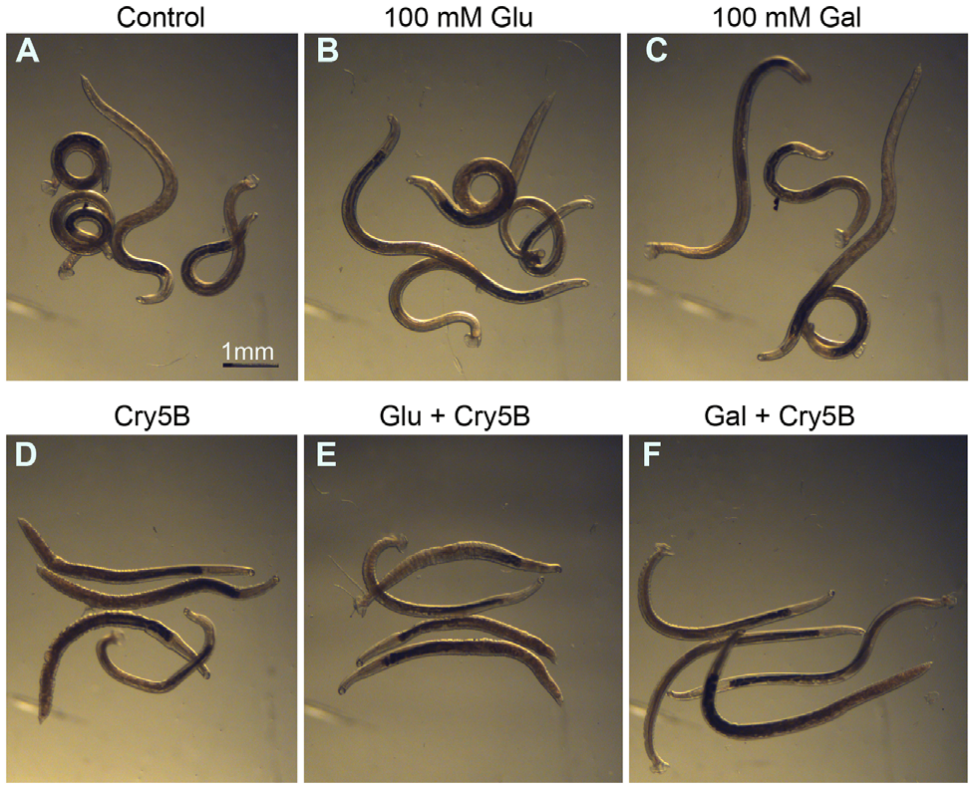
Galactose protects hookworm adults from Cry5B intoxication. All panels are taken at the same magnification after adult *A. ceylanicum* were incubated *in vitro* at the indicated conditions for 48 hr. All compounds were added simultaneously. Shown is one representative experiment (repeated three times). The hookworms in A, B, and C were all highly motile and healthy. The hookworms in F were moderately motile (less motile than the worms in A, B and C, but more motile than the worms in D and E) and looked healthy. The hookworms in D and E were intoxicated as seen by shrinkages in length, shriveled cuticles, and severe decreases in motility. Cry5B was added at 10 µg/mL.

To address whether the mechanism of action of Cry5B in *C. elegans* is conserved in hookworms, we first asked if the functional reliance of Cry5B activity on the invertebrate glycolipid receptor is the same in hookworms as it is in *C. elegans*. This functional reliance was addressed by asking if the toxicity of Cry5B to the parasite can be reduced using galactose, a sugar that competes with Cry5B binding to glycolipid receptors, but not reduced using glucose, a sugar that does not compete with Cry5B binding to glycolipid receptors [22]. We treated *A. ceylanicum* adults *in vitro* with Cry5B alone, Cry5B in the presence of 100 mM glucose, and Cry5B in the presence of 100 mM galactose. In the presence of 100 mM glucose or 100 mM galactose (but no Cry5B), *A. ceylanicum* adults are healthy at 48 hr, similar to no-sugar controls (Figure 1A–C). With the addition of 10 µg/mL Cry5B, *A. ceylanicum* adults become intoxicated, most evident by physical shrinking, ruffling of their cuticle, and loss of motility (Figure 1D). Addition of 100 mM glucose does not alter the ability of Cry5B to intoxicate hookworm adults (Figure 1E) but addition of 100 mM galactose has pronounced protective effects, partly rescuing intoxication in the parasites (Figure 1F). Similar results were found using 50 mM galactose (Figure S2). Thus, galactose, but not glucose, functionally inhibits Cry5B intoxication.

**Figure 2:**
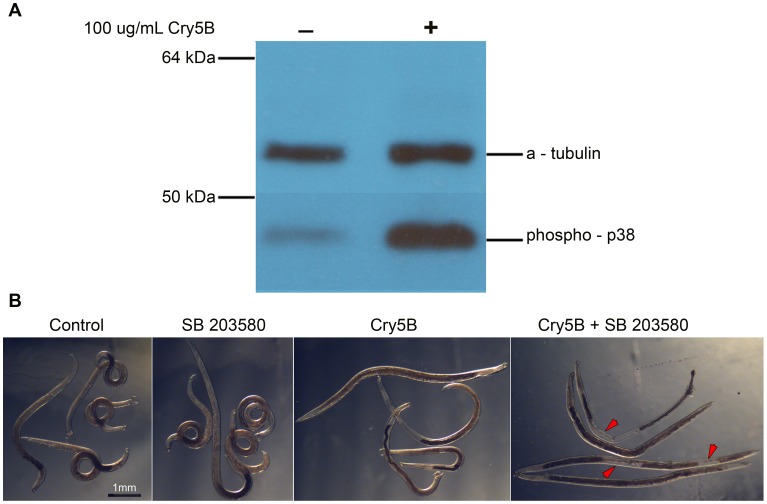
p38 pathway plays an important role in hookworm responses to Cry5B. **A.** *A. ceylanicum* adults (five/condition) were exposed to 100 µg/mL Cry5B for 1 hour, harvested for total protein, and then processed for Western blotting with phospho-p38 antibody. alpha-tubulin antibody serves as a loading control. A large increase in phospho-p38 levels is evident upon addition of Cry5B. **B.** *A. ceylanicum* adults were first incubated in buffer (Control, Cry5B panels) or in buffer plus p38 inhibitor (SB 203580, Cry5B+SB 203580 panels). Twenty-four hours later, Cry5B at 1 µg/mL was added to two of the groups as indicated. Images were taken 96 hr later. Control and SB203580-treated hookworms are healthy. Hookworms treated with 1 µg/mL of Cry5B alone are slightly intoxicated. In the presence of both inhibitor and Cry5B protein, all animals are dead, demonstrating that the p38 pathway protects hookworms against Cry5B.

To test whether the response of *A. ceylanicum* to Cry5B is conserved with the response of *C. elegans* to Cry5B, we performed two experiments. First, we treated *A. ceylanicum* adults and tested for activation of the p38 MAPK pathway. As with *C. elegans* [16], [20], Cry5B treatment of *A. ceylanicum* adults results in markedly increased phosphorylation of p38 MAPK (Figure 2A).

Second, we tested the effect of reducing or eliminating p38 MAPK function on adult hookworms treated with Cry5B. Reduction of p38 MAPK in *C. elegans* results in significant (∼150-fold) increased sensitivity of the roundworm to Cry5B attack [16], [21]. To see if the p38 MAPK pathway also protects hookworm adults from Cry5B attack, we exposed *A. ceylanicum* adults to an inhibitor of the p38 MAPK pathway, SB203580, followed by treatment with a low level (1 µg/mL) of Cry5B. Whereas the parasites are healthy in the presence of inhibitor alone and show only small-moderate levels of intoxication at this low dose of Cry5B alone, treatment of the parasites with Cry5B in the presence of the p38 MAPK inhibitor results in death of 100% of the parasites (Figure 2 B). Thus, the p38 MAPK pathway protects hookworms against intoxication by Cry5B.

### Single-dose *in vivo* efficacy trial of Cry5B against a hookworm infection in hamsters

**Figure 3:**
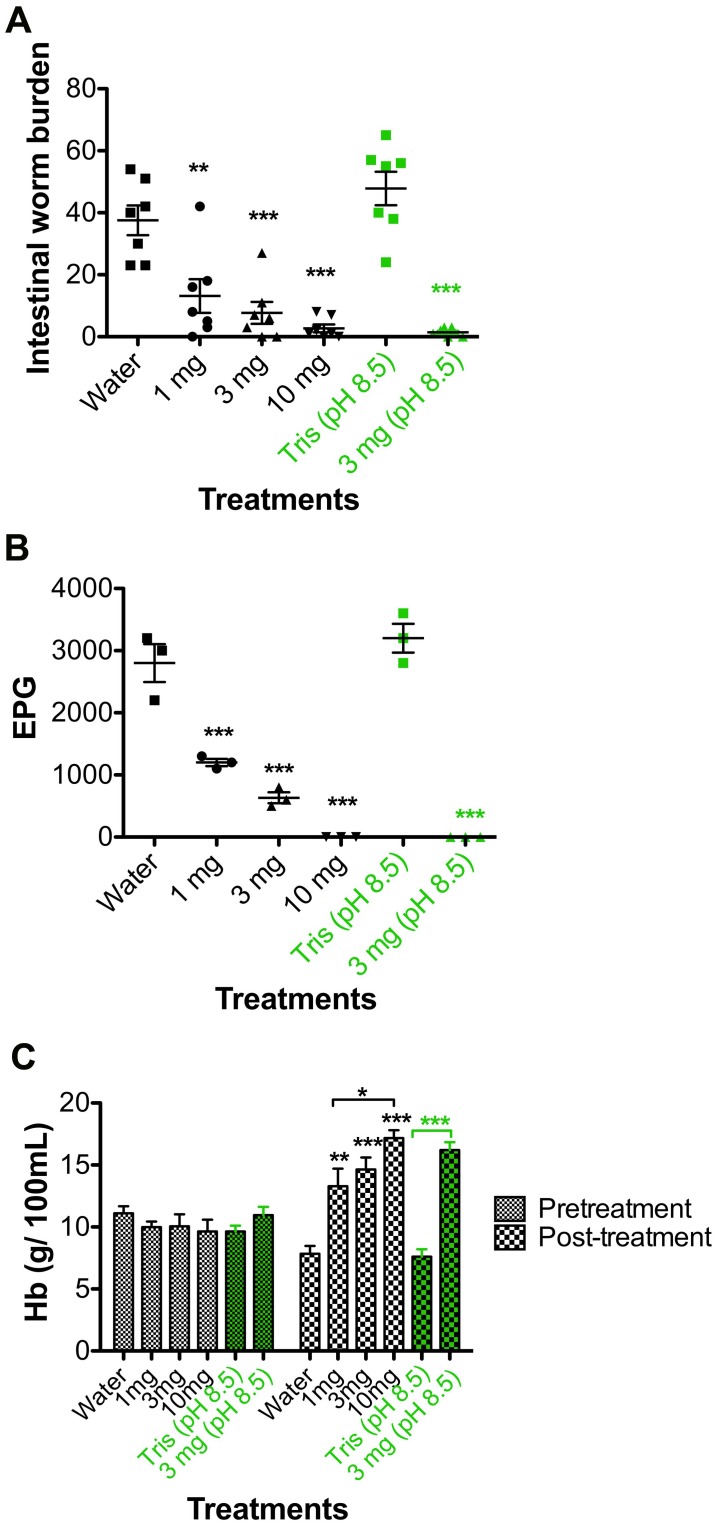
Efficacy of single dose Cry5B against hookworm infection in hamsters. **A. Effects of Cry5B on intestinal worm burdens in** ***A. ceylanicum*** **infected hamsters**. The first four groups (in black, n = 7 per group) shown are the intestinal worm burdens from the groups of infected hamsters treated with a single dose of 1 mg (∼10 mg/kg), 3 mg (∼30 mg/kg), 10 mg (∼100 mg/kg) (715 nmoles/kg) and placebo (ddH_2_O), respectively. The last two groups (in green, n = 7 per group) shown are the intestinal worm burdens of infected hamsters treated with a single dose of 3 mg (∼30 mg/kg) in 150 mM Tris pH 8.5 buffer and placebo (150 mM Tris pH 8.5 buffer). The treatments were conducted on day 16 P.I. and intestinal worm burdens assessed on day 21 P.I. The worm burdens in each hamster are indicated with a separate symbol. Long horizontal bars represent mean worm burdens; smaller bars indicate s.e.m. (standard error of the mean). **B. Effects of single dose Cry5B on egg production in** ***A. ceylanicum*** **infected hamsters.** Shown are the average eggs/gram of feces in each group on day 5 post-treatment. The fecal egg counts in each group are indicated with a separate symbol. Long horizontal bars represent mean eggs per gram of feces (EPG) per group; smaller bars indicate s.e.m. **C. Single dose of Cry5B treatments protects** ***A. ceylanicum*** **infected hamsters from blood loss.** Shown are the hemoglobin levels in each group right before treatment and 5-days post treatment. All values are the means ± s.e.m. In all panels, asterisks indicate statistical significance of placebo *vs.* Cry5B treatment groups. * P<0.05; ** P<0.01; *** P<0.001. No asterisks indicate no difference. For numerical values, please see results section.

Since the accepted method of treatment for hookworm disease worldwide is single-dose mass drug administration (MDA) [4], we tested whether Cry5B could be effective against hookworms *in vivo* at a single dose. Hamsters were inoculated with infectious *A. ceylanicum* L3 larvae and then, on day 16 post-infection (P.I.) when the parasites had reached the adult stage, the hamsters (seven/group) were gavaged with single doses of Cry5B at either 1 mg (∼10 mg/kg), 3 mg (∼30 mg/kg), or 10 mg (∼100 mg/kg). Dose-dependent effects could be seen with parasite reductions of 65%, 79%, and 93% respectively (Figure 3A; relative to water control P = 0.0006, <0.0001, <0.0001 respectively). Strong effects on parasite progeny production were seen with fecal egg count reductions of 57%, 77%, and 100% respectively (Figure 3B; relative to water control P<0.0001 for each). Treatment with single dose of Cry5B significantly protected hamsters from blood loss caused by the hookworm infection. Relative to infected but untreated controls, infected hamsters treated with single-dose of Cry5B (1 mg, 3 mg and 10 mg) displayed significantly higher levels of hemoglobin (Figure 3C; relative to water control P = 0.0006, <0.0001, <0.0001 respectively). Furthermore, Cry5B treatment resulted in significantly improved hemoglobin levels over time relative to pre-treatment levels, whereas placebo controls did not show improvement (Figure S3).

### Buffering Cry5B increases efficacy

Since Cry5B rapidly degrades in simulated stomach fluid (>99% of the protein is digested in 4 minutes [14]), we hypothesized that neutralization of stomach acid would inhibit the activity of pepsin, decrease the digestion of Cry5B in stomach, and improve its anthelmintic efficacy. We therefore, simultaneous with the above experiments, included two additional groups: one in which we gavaged infected hamsters with 150 mM Tris pH 8.5 buffer as control and another in which we gavaged 3 mg Cry5B in 150 mM Tris pH 8.5. The purpose of the basic buffer was to fully or partially neutralize stomach acid. Whereas, relative to water controls, pH 8.5 buffer alone did not affect worm burdens (P = 0.45), fecal egg count (P = 0.52), or hemoglobin levels (P = 0.95), 3 mg (∼30 mg/kg) of Cry5B gavaged in pH 8.5 buffer showed dramatic improvements on reducing hookworm burden and hookworm disease relative to either placebo control (water or pH 8.5 buffer). Relative to pH 8.5 buffer alone, 3 mg Cry5B in pH 8.5 buffer results in 97% reduction in intestinal worm burden, 100% reduction in fecal egg counts, and significant improvement in hemoglobin levels (Figure 3A, B, C; P≤0.0001 for each).

Gavage of 3 mg Cry5B in pH 8.5 buffer showed better efficacy than gavage of 3 mg of Cry5B in water relative to worm burdens (97% reduction vs. 79% reduction), fecal egg counts (100% reduction vs. 77% reduction), and hemoglobin levels (16.2 vs. 14.6 g/100 ml). A direct comparison between results with 3 mg Cry5B in water and 3 mg Cry5B in pH 8.5 buffer indicate that the additional decrease in intestinal worms burdens is just out of the range of statistical significance (P = 0.064; 1-tailed T-test unequal variances), but that the additional difference in fecal egg counts is statistically significant (P = 0.0094; 1-tailed T-test unequal variances). The level of disease reduction seen with 3 mg Cry5B in pH 8.5 buffer was similar to levels seen with 10 mg Cry5B treatment in water (unbuffered), suggesting that this method of neutralizing stomach acid results in ∼3-fold increase in efficacy.

## Discussion

We demonstrate here the functional conservation of Cry5B action against the hookworm *A. ceylanicum* and the free-living nematode *C. elegans*. Co-incubation of hookworms with Cry5B and galactose, which in *C. elegans* competes with Cry5B to binding to the glycolipid receptors, reduces Cry5B activity in *A. ceylanicum* as it does in *C. elegans* [22]. Furthermore, Cry5B treatment induces activation of p38 MAPK in hookworms as it does in *C. elegans* [16], [20]. Strikingly, inhibition of the p38 MAPK pathway in hookworms results in increased activity of Cry5B, as it does in *C. elegans* [16], [21]. To our knowledge, this finding represents the first time that experimental inhibition of a hookworm pathway results in increased activity of an anthelmintic.

Our results suggest that invertebrate-specific glycolipid receptors are functionally important for Cry5B activity in hookworms as they are in *C. elegans*. This finding is important since these receptors are not found in vertebrates and since it provides a molecular basis of Cry5B safety in vertebrates.

Furthermore, our results indicate that key knowledge about how *C. elegans* responds to Cry5B (*i.e.*, via p38 MAPK) applies to how hookworms respond to Cry5B. The conservation of functional action with *C. elegans* is important since there is a wealth of information about how *C. elegans* responds to Cry5B action, including more than 100 genes that, when knocked down, result in hypersensitivity of *C. elegans* to Cry5B [16], [17], [18], [19], [20], [27]. Interestingly, of these more than 100 hypersensitivity genes, four of them are conserved in nematodes (including parasites) but lacking in vertebrates [16]. Given our results, which indicate that Cry5B molecular response pathways in *C. elegans* can apply to hookworms, then these genes represent targets for knock-down by RNAi or drug development that would safely and significantly potentiate Cry5B action. This approach represents a forward-design for synergistic anthelmintic therapy. Instead of looking for synergy amongst existing drugs, a treatment could be developed specifically to produce a synergistic effect by inhibiting genes involved in roundworm innate protection against Cry5B.

We also demonstrate here that single dose Cry5B can produce a near complete cure of *A. ceylanicum* hookworm infections in hamsters. Doses of either 10 mg (∼100 mg/kg) in water and 3 mg (∼30 mg/kg) in pH 8.5 buffer results in 93 and 97% reductions in worm burdens respectively and in complete elimination of parasite egg production.

Furthermore, use of pH 8.5 buffer versus water significantly improved the ability of Cry5B to eliminate parasite egg production and resulted in lower parasite burdens. Our results using a basic buffer to deliver better efficacy are consistent with neutralization of stomach acid and pepsin digestion as being important for optimization of Cry5B therapy. With regards to therapeutic application, our results suggest that the absence of a period at low pH leads to greater retention of Cry5B activity. In practice, this could be achieved by encapsulation of the protein during the passage through the stomach to protect it from acid (*e.g.*, by applying a simple, cheap enteric coating to a capsule containing Cry5B).

Our results compare favorably with another new anti-hookworm drug under development, K11777, which showed 90% clearance of parasites at a dose of 100 mg/kg (compared to 97% clearance for Cry5B at ∼30 mg/kg, which, on a molar level is <800X that of K11777 [28]). Cry5B is predicted to be very safe for human therapy as Cry proteins, famous for their use as insecticidal proteins in sprays and transgenic crops, are generally considered non-toxic to vertebrates and as the receptor for Cry5B in roundworms are invertebrate-specific glycolipids (this work and [11], [22], [29]).

In summary, our results demonstrate that Cry5B can be a highly effective single-dose therapy for *A. ceylanicum* hookworm infections in hamsters (97% parasite clearance), that inclusion of a basic buffer increases the therapeutic activity of the protein, that Cry5B - invertebrate-specific glycan interactions are apparently important for Cry5B action in hookworms, and that the key p38 MAPK Cry5B defense pathway, which operates in *C. elegans*, also operates in hookworms. These results advance the development of Cry5B as a novel, safe, highly-effective single-dose therapy against hookworms and potentially many other intestinal roundworm infections.

The sequence of Cry5B can be found via the European Bioinformatics Institute website, accession number U19725.

Supporting Information includes one table and three figures that present the numerical data for: 1) Table S1: the *in vivo* curative experiment, 2) Figure S1: the hamster body weights for the curative experiment, 3) Figure S2: the *in vitro* galactose rescue experiment using a dose of 50 mM galactose, and 4) Figure S3: an alternative way of looking at the hemoglobin levels in the curative experiment, comparing hemoglobin levels pre- and post-treatment for each group.

## Supporting Information

Figure S1 **Body weight of the hamsters in each experimental group before treatment.** Shown is the average body weight in each experimental group (n = 7 in each group) just prior to treatment. Error bar is the standard error of mean.
(TIFF)

Figure S2 **50 mM galactose protects hookworm adults from Cry5B intoxication.** All panels are taken at the same magnification after adult *A. ceylanicum* were incubated *in vitro* at the indicated conditions for 96 hr. All compounds were added simultaneously. Shown is one representative experiment (repeated three times). The hookworms in the control group and the 50 mM galactose group were all highly motile and healthy. The hookworms in the galactose plus Cry5B group are motile, although less so than in the control group. The hookworms in the Cry5B only group are all dead.
(TIF)

Figure S3 **Relative to levels before treatment, Cry5B treatment results in significantly improved hemoglobin levels whereas placebo controls do not show improvement in hemoglobin levels.** A one-tailed Student's t-test (assuming improvement in hemoglobin levels) was used to compare data within each group. In all paired columns, asterisks indicate statistical significance of pre-treatment (pre-) *vs* post-treatment (post). * P<0.05; ** P<0.01; *** P<0.001. No asterisk indicates no difference.
(TIFF)

Table S1 **Numerical data of worm burdens, fecal egg counts and hemoglobin levels for Cry5B** ***in vivo*** **treatments experiments.**
(DOC)
