# Supplementary figures and images for: Mechanistic and Single-Dose In Vivo Therapeutic Studies of Cry5B Anthelmintic Action against Hookworms
Source: PLoS Negl Trop Dis. 2012 Nov 8;6(11):e1900. doi: 10.1371/journal.pntd.0001900 (PMC3493396; doi:10.1371/journal.pntd.0001900)

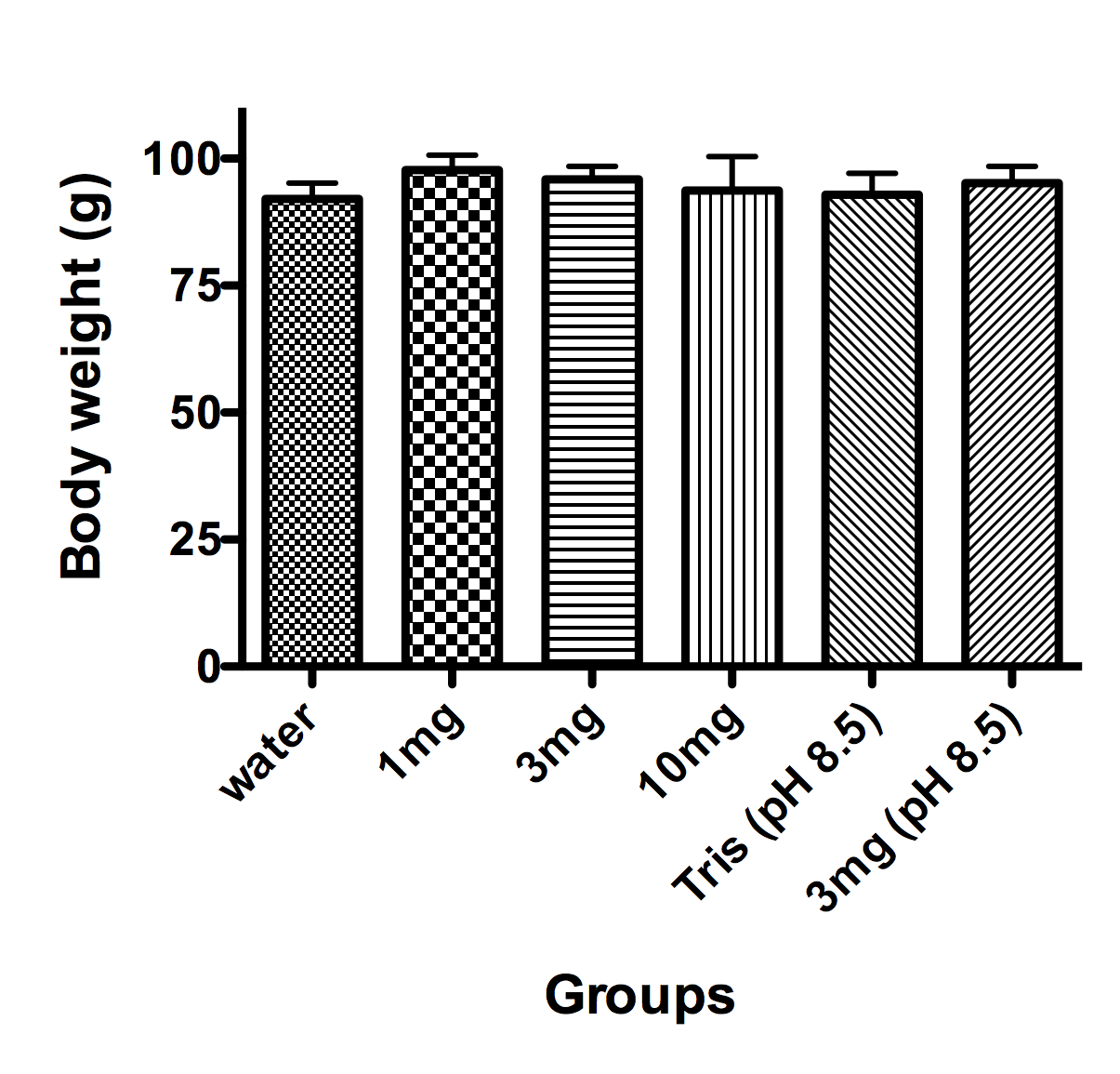

Supplement: Figure S1 — Body weight of the hamsters in each experimental group before treatment. Shown is the average body weight in each experimental group (n = 7 in each group) just prior to treatment. Error bar is the standard error of mean. (TIFF) [file pntd.0001900.s001.tiff]

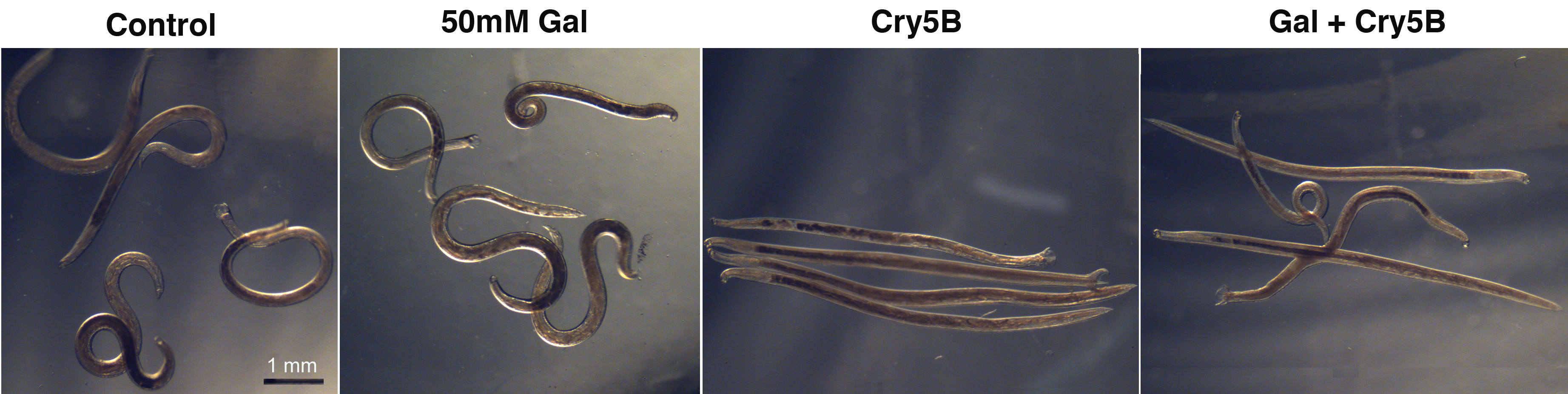

Supplement: Figure S2 — 50 mM galactose protects hookworm adults from Cry5B intoxication. All panels are taken at the same magnification after adult A. ceylanicum were incubated in vitro at the indicated conditions for 96 hr. All compounds were added simultaneously. Shown is one representative experiment (repeated three times). The hookworms in the control group and the 50 mM galactose group were all highly motile and healthy. The hookworms in the galactose plus Cry5B group are motile, although less so than in the control group. The hookworms in the Cry5B only group are all dead. (TIF) [file pntd.0001900.s002.tif]

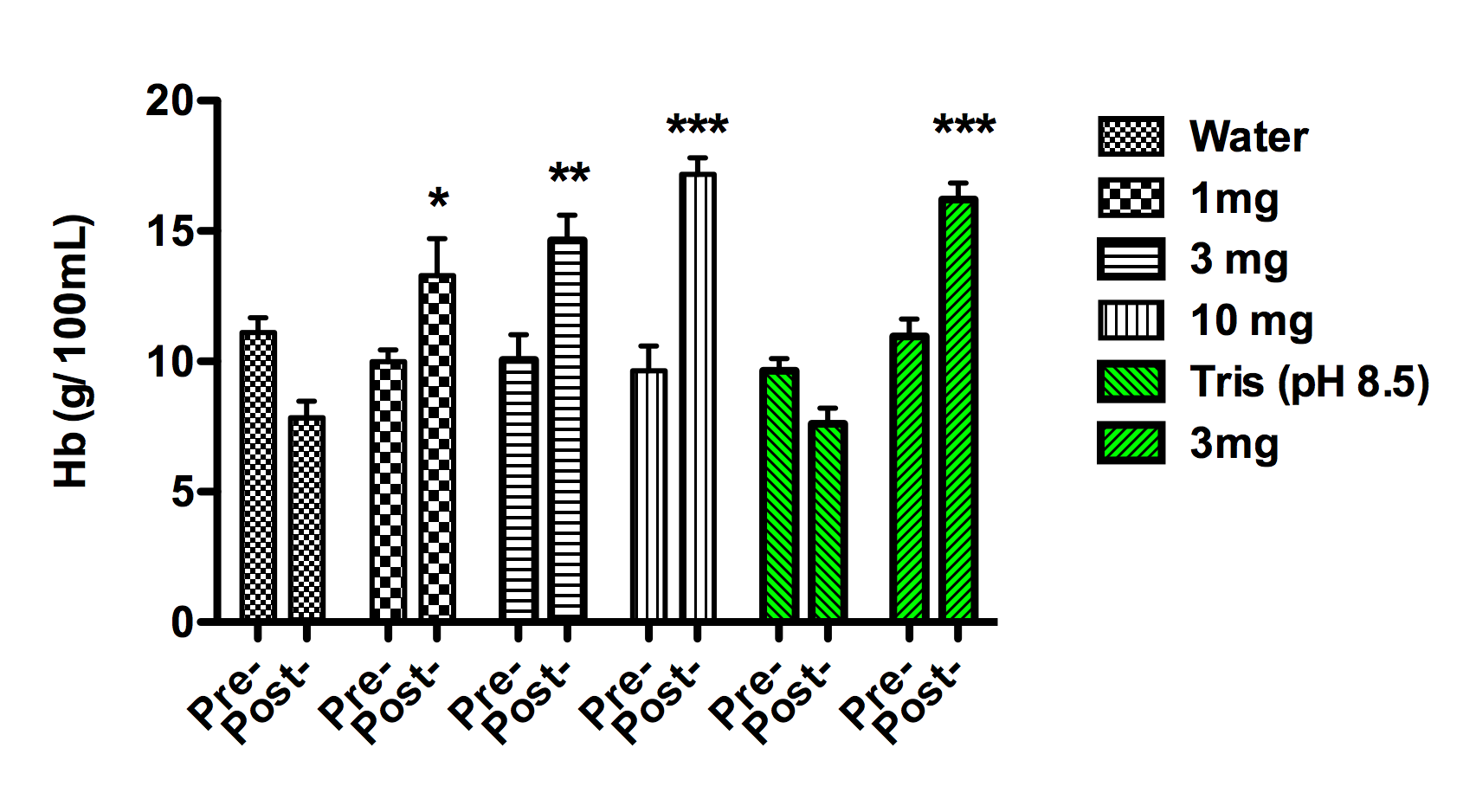

Supplement: Figure S3 — Relative to levels before treatment, Cry5B treatment results in significantly improved hemoglobin levels whereas placebo controls do not show improvement in hemoglobin levels. A one-tailed Student's t-test (assuming improvement in hemoglobin levels) was used to compare data within each group. In all paired columns, asterisks indicate statistical significance of pre-treatment (pre-) vs post-treatment (post). * P<0.05; ** P<0.01; *** P<0.001. No asterisk indicates no difference. (TIFF) [file pntd.0001900.s003.tiff]

**
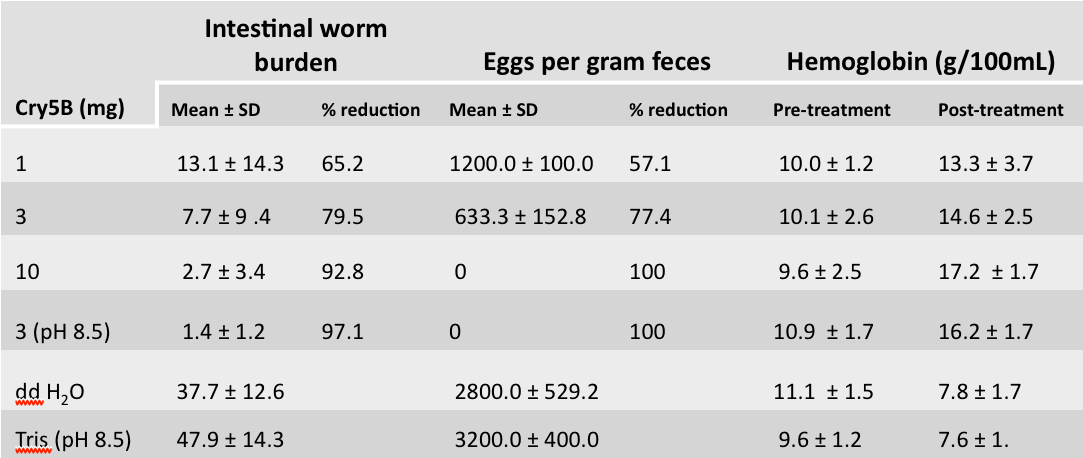
**

Supplement: Table S1 — Numerical data of worm burdens, fecal egg counts and hemoglobin levels for Cry5B in vivo treatments experiments. (DOC) [file pntd.0001900.s004.doc]
